# Development and reliability testing of the Scale for the Evaluation of Staff‐Patient Interactions in Progress Notes (SESPI): An assessment instrument of mental health nursing documentation

**DOI:** 10.1002/nop2.254

**Published:** 2019-03-21

**Authors:** Kjellaug K. Myklebust, Stål Bjørkly

**Affiliations:** ^1^ Faculty of Health Sciences and Social Care Molde University College Molde Norway; ^2^ Centre for Forensic Psychiatry Oslo University Hospital Oslo Norway

**Keywords:** empathy, inpatients, instrument development, medical record, nurse–patient relations, nursing assessment, psychiatric nursing

## Abstract

**Aim:**

To develop and test the reliability of the Scale for the Evaluation of Staff‐Patient Interactions in Progress Notes (SESPI). Therapeutic nurse–patient interactions are fundamental in mental health nursing. However, little is known about how these interactions are recorded in nursing documentation and there is no instrument available for collecting this type of information for quantitative analysis.

**Design:**

Instrument development and reliability testing.

**Methods:**

The development of the SESPI was based on qualitative analyses of progress notes retrieved from patient records in two mental health services. A self psychological attunement perspective guided the analyses. SESPI was tested for internal consistency and inter‐rater reliability after 22 nurses independently scored 10 progress notes.

**Results:**

Cronbach's alpha for the entire instrument was 0.977, indicating that the raters' scores had very high internal consistency. ICC was 0.770. The alpha and ICC values for each step were high, varying between 0.970 and 0.992.

## 1 INTRODUCTION

This article reports on the development of an instrument for assessing the quality and extent of reported interactions between psychiatric ward staff and patients in nursing documentation. Ward staff comprises professionals with different educational backgrounds such as, for instance, registered nurses, social workers and healthcare assistants. All of them write progress notes as part of their daily nursing documentation. Hereafter, the term *staff* will refer to all the professional ward staff.

The importance of the therapeutic relationship is emphasized in mental health nursing literature (e.g. Owens, Haddock, & Berry, 2013; Wheeler, 2011). Empathy is vital in a therapeutic relationship (Bee et al., 2008; Peplau, 1952; Travelbee, 1963). Though the importance of the therapeutic relationship to psychotherapeutic outcome has been established for years (Norcross & Lambert, 2018), therapeutic relationship as well as empathy have been difficult to operationalize and measure in nursing contexts (McAndrew, Chambers, Nolan, Thomas, & Watts, 2014; Yu & Kirk, 2008, 2009). Nearness and distance between persons wax and wane. A relationship between two persons reflects how they and others perceive them as a pair. Interactions between persons are more concrete and easier to observe and report. Mental healthcare staff have emphasized the importance of empathy in conflict management, and empathic communication is associated with the reduction in seclusion and restraint. However, there is a debate about the precise meaning of empathy and how staff can improve their empathic skills (Gerace, Oster, O'Kane, Hayman, & Muir‐Cochrane, 2018; Yu & Kirk, 2008).

Heinz Kohut postulated that empathy and attunement are core aspects in self psychological psychotherapy and empathy pre‐supposes attunement (Kohut, 1977, 1984; Rowe & Mac Isaac, 1989). To attune to someone emotionally means to seek to understand the emotional and relational basis of another's behaviour rather than just the actual behaviour (Erskine, 1998). *Positive attunement* is the process of trying to come as close as possible to apprehending another person's subjective experience and communicating this understanding to her. This is one of the core features of empathic therapeutic interaction. Current psychotherapeutic literature highlights the significance of empathy and attunement in therapeutic relationships (Cooper, 2008; Finlay, 2016). The relevance of attunement goes beyond the psychotherapy context; for instance, Delaney, Shattell, and Johnson (2017) pointed to attunement as an essential nursing skill for creating therapeutic relationships in inpatient psychiatric care. Moreover, Lorem and Hem (2012) emphasized the importance of an attuned understanding in the relationship between staff and patients suffering from psychosis.

Considering the strong emphasis on the importance of the therapeutic professional–patient relationship in mental health practice, it is highly relevant that the quality and extent of staff–patient interactions be described in nursing documentation. To the authors' knowledge, no instrument exists for collecting this type of information for quantitative analysis. We searched in Cinahl, Medline, PsycInfo and ProQuest with the combinations of terms shown in Columns 1 and 2 of Table 1.

**Table 1:** Terms used in search for literature

1	2
Nursing/staff: ‐ Record ‐ Care planning ‐ Documentation ‐ Note ‐ File ‐ Progress note Electronic health record Medical record systems	Scale Instrument Tool Measure

Systematic reviews have identified a range of audit instruments for nursing documentation, but not one had explicit measures for staff–patient interactions or for attunement and empathy (Saranto & Kinnunen, 2009; Wang, Hailey, & Yu, 2011). For studies measuring the presence of staff–patient interactions in nursing documentation, we found only one quantitative study: Juvé‐Udina et al. (2014) evaluated the frequency of documented psychosocial interventions in acute care settings, but only 3.8% of the data material were collected in mental health services. Qualitative studies from different mental health contexts found that nursing documentation primarily comprised nurses' observations of patients' behaviour and provided only limited information about staff–patient interactions (Buus & Hamilton, 2016; Martin & Street, 2003). This finding was confirmed in interviews with staff working in acute and open mental health services. The staff focused on documenting observations of patients for diagnostic purposes rather than on staff–patient interactions. Neither challenging interactions that succeeded in attuning to patients nor communications that failed to meet patients' emotional needs were reported (Myklebust, Bjørkly, & Råheim, 2018).

This article describes a reliability study of a scale that was developed to assess therapeutic staff–patient interactions recorded in nursing documentation. The scale was developed drawing on an analysis of a selection of staff progress notes. Progress notes in mental health services are the staff's reports in the electronic patient records that are usually written for every shift. Although some may question whether progress notes actually provide an accurate picture of staff–patient interactions, these notes are, nonetheless, the best accounts available short of doing observational studies (which involve very challenging practical and ethical issues). The following is an example of a progress note used in the current study (context: the patient was under mechanical restraint):

Anne was very anxious and agitated. When the nursing staff tried to talk to her, she strongly rejected contact and her body language expressed contempt and fury. Another nurse approached her calmly. She took her hand and tried, without saying anything, to show interest and care. The patient calmed down quickly and the nurse gave her time to express and verbalise her sad and angry feelings.

This is a straightforward example of a report that demonstrates how successful attunement between a patient and a professional opened up a dialogue and how progress notes can provide valuable data for evaluating therapeutic relationships in mental health services. The instrument developed in the current study was theory‐driven, inspired by Kohut's emphasis on attunement in self psychology. Thus, the analysis of progress notes in this study focused on text describing patients' experiences and staff's attunement to the described experiences. The aim of the study was to develop and test the reliability of the Scale for the Evaluation of Staff‐Patient Interactions in Progress Notes (SESPI).

## 2 METHODS

### 2.1 Design and data collection

The progress notes used for developing the SESPI were retrieved from 10 electronic patient records from an acute psychiatric ward and 10 from an open inpatient unit in a district psychiatric centre in Norway. There were 12 beds on the acute ward and 14 beds at the district psychiatric centre, with a total of about 90 staff members writing progress notes. Electronic documentation was implemented 15 years ago in these hospital units. These two wards were chosen to obtain progress notes from different contexts regarding severity of psychotic symptoms and challenging behaviours, involuntary versus voluntary admission and treatment with or without use of coercion and seclusion. The patients (*N *=* *20) gave their consent to use their anonymous records for this purpose.

To avoid bias in the selection of progress notes, the study had a retrospective design. We used only progress notes written before the staff were informed about this research. Staff recruited patients who had been admitted to the wards any time during a 6‐month period from November 2015, using the following criteria:

- The patient had been admitted to the ward at least once in the period between 1 November 2013 and 1 November 2015 (staff were informed about the study on the last‐mentioned date).
- One of the admission periods had lasted at least 14 days.

After a patient consented to take part in the study, a nurse from each ward retrieved progress notes from the patient's electronic record. If the patient had been hospitalized more than once during the identified period, the most recent admission was selected. Progress notes were retrieved from admittance until discharge with an upper limit of 4 weeks.

### 2.2 Instrument development

Below is a description of the process entailed in constructing the SESPI scale. The progress notes were prepared for analysis using the following procedure:

- Descriptions of all types of communication between staff and others, without the patient's being present in the actual situation, were removed.
- Notes from conversations between patients and those other than the staff (e.g. psychiatrists, family, friends) were removed.

Thereafter, after the above procedure, the remaining content of the progress notes was termed “an excerpt.” The excerpts described episodes where staff and patients had an opportunity to interact. The development of the SESPI was based on qualitative analyses of these excerpts. The attunement perspective guided the analysis. The first author collected and grouped text describing patients' experiences after which both authors developed adequate quality category labels for the described experiences. Texts describing staff–patient interactions were analysed accordingly.

In total, 1050 excerpts were retrieved from the electronic patient journals and all were included in the qualitative analysis for scale development. Five categories describing patients' experiences and five types of staff–patient interactions emerged from the analysis, including a neutral category for experiences and a neutral category for attunement. A selection of excerpts was scored to test these categories. In the process, we realized that the categories Neutral experiences and Neutral attunement were not part of what we were aiming to measure and analyse and thus excluded them. Consequently, the scale ended up with four categories for patient experiences and four types of attunement. Adjustments to the scale were made until the first and second authors were able to score the 1051 excerpts consistently, based on independent judgement and consensus.

### 2.3 The raters

A sample of 22 raters (1 man, 21 women) participated in the reliability testing of the SESPI in October 2016. According to Bujang and Baharum (2017), this is the sample size required for the 10 observations (excerpts) the raters had in our study (statistical power of 80%, alpha = 0.05 and an effect size difference of ICC = 0.20). We think that a detection of an effect size difference of 0.20 (ICC = 0.70 vs. 0.50) is acceptable for testing instrument development. Raters' ages ranged from 25 to 71 years old. Seventeen were registered nurses, and the remaining five had bachelor's degrees in social or health care‐related work. The raters' experience with writing progress notes in a mental health service context varied from 1 to 20 years (*Mean *= 5.95, *SD *= 4.85). Sixteen were recruited through a continuing education programme in mental health at Molde University College in Norway. In addition, six nurses, previously graduated from the above‐mentioned programme, volunteered to participate. Testing of the scale for the 16 students took place in a 90‐min break between lectures at the University College. The procedure for the introduction and testing of SESPI was the same for the group of six nurses, but the scoring session took place at one of their regular meetings for reflection on work‐related issues.

### 2.4 Procedure

Ten excerpts for the reliability testing of the SESPI were picked from the pool of excerpts retrieved in the qualitative investigation of progress notes from the locked acute and open psychiatric wards. The selection of the excerpts was guided by the intention to test all four steps in the SESPI. Accordingly, the 10 excerpts represented a variety concerning if and to what extent, they contained descriptions of patients' experiences and staff's approaches. Before further description of the procedure for the reliability testing, we will present the SESPI.

#### 2.4.1 The instrument SESPI

**Figure 1:**
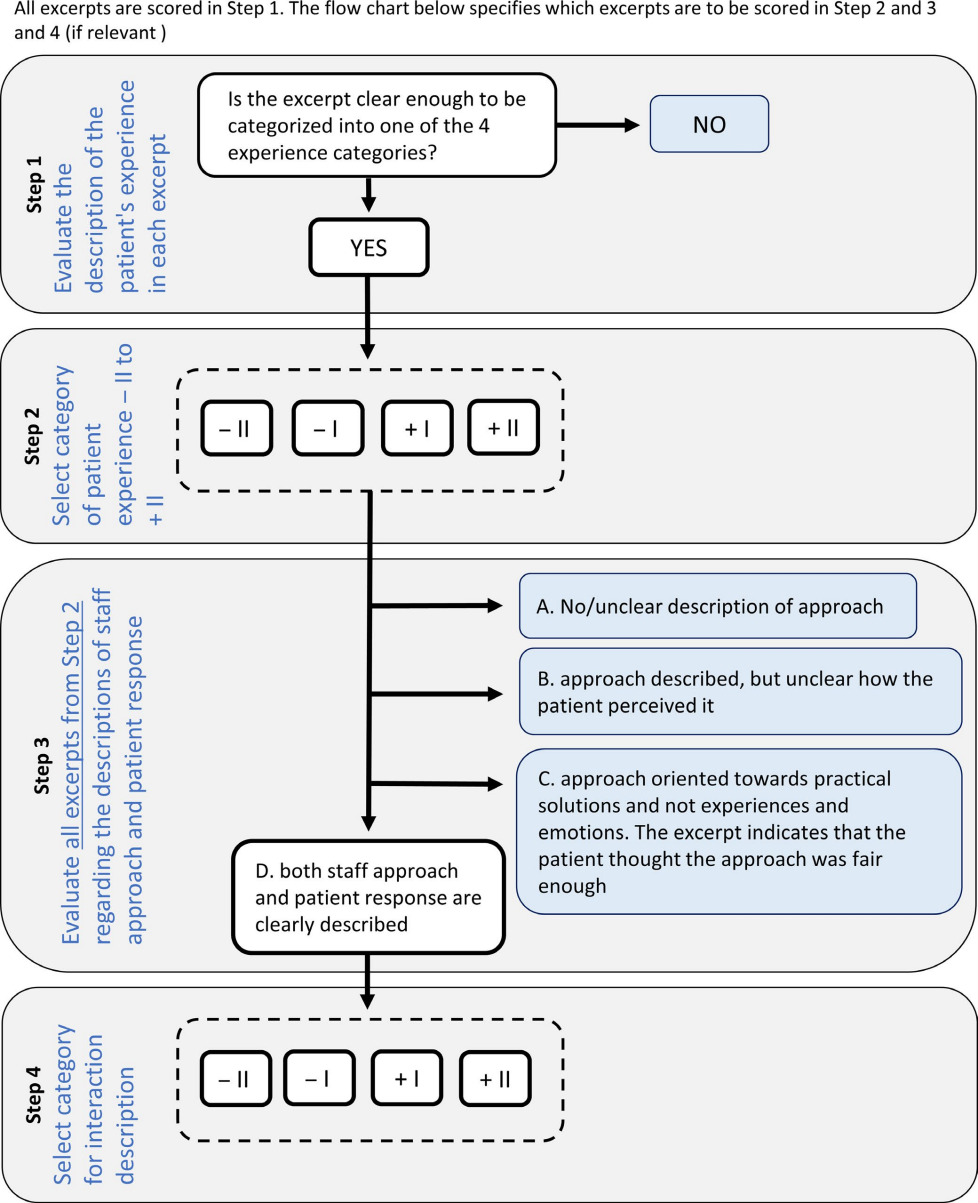
Flow chart for scoring excerpts in SESPI

The SESPI consists of four coding steps (Figure 1). In Steps 1 and 3, excerpts that lack a description of patient's experience or the staff's attunement are excluded. Step 1 sorts out whether an excerpt contains a description of the patient's experience or not. To pass Step 3, there must be an account of both the patient's expressed experience and the involved nurse's attunement to it. Steps 2 and 4 include categories to assess the *quality* of patients' experiences and staffs' attunements, respectively.

In Step 1, all excerpts are scored dichotomously: “Does the excerpt contain some kind of description of patient's experience?” A *yes* means proceed to Step 2; a *no* signifies no further assessment of that excerpt. A typical example that would be scored “no” is “Mostly stayed in his room. He answers briefly when the staff poses specific questions.”

The following excerpt describing the interaction between staff and Anne (cited earlier) will be used to illustrate how excerpts were scored in the SESPI:

Anne was very anxious and agitated. When the nursing staff tried to talk to her, she strongly rejected contact and her body language expressed contempt and fury. Another nurse approached her calmly. She took her hand and tried, without saying anything, to show interest and care. The patient calmed down quickly and the nurse gave her time to express and verbalise her sad and angry feelings.

In Step 1, this excerpt was scored “yes,” and thus, the scoring proceeded to the next step.

Step 2 has four possible scores of patient experiences:

‐ II = Very uncomfortable
‐ I = Uncomfortable
+ I = Positive
+ II = Very positive

Distinctions between ‐ II, very uncomfortable and ‐ I, uncomfortable, are determined by intensity and duration. An acute anxiety attack indicates a very high level of discomfort and intensity and belongs to category ‐ II. Thus, the excerpt above, where Anne is described as *“*very anxious and agitated” and her body language expressed “contempt and fury,” was scored ‐ II Very uncomfortable. Long‐lasting experiences (duration) are similarly scored: for example, a patient might repeatedly complain (not necessarily powerfully), throughout an entire shift, that all hope is gone. This, too, would be scored ‐ II Very uncomfortable.

The positive + II experiences have greater importance and are longer lasting than + I experiences. Category + I does not require a positive experience in the “big picture,” but, rather, a positive experience in the moment (e.g. *he expressed relief to be admitted to the ward*). An example of an excerpt scored as + II is as follows:

She was writing in her diary about her life before she was hit by the accident and how she feels right now. She says she has not been able to write in 10 years and she is excited over this rediscovery.

In Step 3, the descriptions of staff–patient interactions are assessed by four alternatives:

A. No or insufficient description of the staff's approach.
B. The staff's approach is described, but the patient's response is not, or not sufficiently, described.
C. The staff's approach is described, and the patient appears to experience it as fair enough. However, the approach is mostly oriented to practical solutions and fails to grasp the patient's experience or feelings.
D. Both staff's approach and patient's response are described.

Our example with Anne and the staff was scored D. However, if this excerpt had stopped at “her body language expressed contempt and fury,” the correct score would be A (no description of staff's approach). Finally, if the excerpt described that the nurse “took her hand and tried, without saying anything, to show interest and care,” but failed to describe the patient's response, alternative B would be correct.

An example of an excerpt scored in category C, Step 3, was the following:

He was anxious and asked for and was given anxiolytic medicine. After a while, he said he felt less troubled.

This description indicates that the man experienced the nurse's approach as appropriate, but, on the other hand, the nurse did not invite him to express his experiences. Thus, this could not be scored as a category D and only excerpts scored as D (“both staff's approach and patient's response are described”) proceed to Step 4.

Step 4 contains four quality categories of attunement in the staff–patient interactions:

‐ II = Failed attunement
‐ I = Partially failed attunement
+ I = Partially successful attunement
+ II = Successful attunement

Excerpts where the staff member is described as being in an excessively expert position and the patient's response is strongly negative belongs to ‐ II. For example:

He was restless and could not sleep. He wanted a vitamin supplement, but his request was rejected with the explanation that it was the middle of the night and that he had better get some sleep. He slammed the door loudly and shouted: “I want it now!”

Category ‐ I includes a continuum from slightly authoritative approaches to approaches where the staff have tried to grasp the patient's experience, but the patient's reaction was negative.

The + I and + II categories include both approaches to explore or confirm a patient's experience and positive feedback from the patient. The following is an example of an excerpt that was scored as + I:

He was upset after the phone call with his mother. The contact nurse invited him along for a road trip and a walk. He came along and it seemed to ease his worries and to be a help for him to handle his difficult thoughts.

This description is imprecise, not describing, for instance, any questions the nurse asked. In contrast, a + II rating requires exact descriptions of a nurse's intervention, as in our excerpt with Anne where the nurse:

approached her calmly. She took her hand and tried, without saying anything, to show interest and care. The patient calmed down quickly and the nurse gave her time to express and verbalise her sad and angry feelings.

For more examples, see the SESPI (Appendix S1).

The SESPI and a short guideline were e‐mailed to the raters before the reliability testing. The guideline contained some test samples of excerpts and how to code them. The raters were given a 20‐min oral introduction to the SESPI. Each rater completed scoring five excerpts for practice, and then, the first author presented correct scores to all participants. Finally, the raters received 10 copies of the SESPI and 10 excerpts, both numbered from 1 to 10. They received the following instructions before they scored the excerpts independently:

Use copy 1 of the SESPI to code excerpt 1, copy 2 to code excerpt 2 in SESPI, etc.
All of the 10 excerpts are to be scored in Step 1 of the SESPI.
Use the flow chart, Figure 1, to determine whether the actual excerpt is to be scored further in Steps 2 and 3 and 4.

The SESPI has 14 score alternatives per rater, for each report excerpt (see Figure 1). A correct score in Step 4 depends on correct scores in both Step 1 and 3 (one has to score “yes” in Step 1 and alternative “D” in Step 3 to proceed to Step 4). Accordingly, there is only a 3.1% probability of making a correct score by chance in Step 4: Step 1: ½ (one out of two choices) × Step 3: ¼ × Step 4: ¼ = 1/32 (3.1%). Altogether, the total score range number was 3,080 (22 raters × 10 excerpts × 14 alternatives per excerpt = 3,080). This provided a substantial variance for the inter‐rater reliability analysis.

### 2.5 Statistical analyses

Two tests of scale reliability were used. We used Cronbach's alpha to estimate the internal consistency of the scores on the SESPI total and for Steps 2, 3 and 4 separately. Kuder–Richardson 20 was used for the dichotomous scale in Step 1. The Intraclass correlation coefficient (ICC) was chosen to calculate the inter‐rater reliability of the distribution of scores for the 10 excerpts across the four steps of coding and for Steps 1, 2, 3 and 4 separately. The Two‐way Random effects model was used because both raters' and items' (the excerpts) effects were considered random, and our aim was to generalize our reliability results to other mental health staff. We used absolute agreement for estimating ICC for SESPI total and each step of the scale because the purpose of this investigation was to estimate systematic variability due to raters. A conventional 5% significance level and 95% confidence interval (CI) were used. Statistical analyses were conducted using the Statistical Package for Social Science (SPSS) Version 24.

### 2.6 Ethical considerations

The Regional Committee for medical and health research ethics in Norway approved the study on the condition that patients consented to the use of the progress notes in their journals (reference number 2015/1471). All personal identifying information in the progress notes was removed before copies were given to the researchers.

## 3 RESULTS

### 3.1 Inter‐rater and internal consistency reliability of SESPI estimated for the entire instrument

The SESPI was tested regarding inter‐rater reliability and internal consistency. Cronbach's alpha for instrument total = 0.977. This is a very high alpha value, indicating that raters' scores had very high internal consistency for the four steps of the SESPI. Regarding the distribution of scores for the excerpts in each of the four steps, the ICC was lower, but still acceptable; ICC = 0.770 (95% CI 0.608–0.888).

### 3.2 Inter‐rater and internal consistency reliability for each step of SESPI

Step 1 has two score alternatives, *yes* or *no*. The internal consistency reliability was very high (Kuder–Richardson 20 for Step 1 = 0.984). The absolute inter‐rater agreement for all scores (*N *=* *10 scores per rater) was very high (95.9%, see Table 2). ICC for Step 1 was 0.982 (95% CI 0.959–0.995); however, this result must be interpreted with cautiousness because Step 1 is a dichotomous scale.

**Table 2:** Percentage inter‐rater agreement, steps 1–4

	Mean absolute score agreement	Mean absolute score agreement
Step 1 *N* = 22	95.9	
Step 2 *N *= 22	76.8	94.5^a^
Step 3 *N *= 22	84.1	
Step 4 *N *= 22	66.4	89.8^b^
Mean	80.8	92.2

^a^Main category for Step 2 = Positive *or* negative patient experience reported. ^b^Main category for Step 4 = Positive or negative staff attunement *and* positive or negative patient response reported (Step 4).

Steps 2 through 4 have a 4‐point continuous scale for each step. Cronbach's alpha and the ICC values for these three steps were very high. Cronbach's alpha for Step 2 was 0.992, ICC = 0.992 (95% CI 0.981–0.998). The absolute agreement for all 10 excerpts' scores in Step 2 was 76.8%. The choices were ‐ II Very uncomfortable, ‐ I Uncomfortable, + I Positive and + II Very positive. The agreement was 94.5% when merging category ‐ II and ‐ I versus + I and + II (see Table 2). Cronbach's alpha for Step 3 was 0.970 and ICC was 0.968 (95% CI 0.929–0.991). The percentage agreement was high (84.1) (see Table 2). Cronbach's alpha for Step 4 was 0.981 and ICC was 0.978 (95% CI 0.950–0.994). Step 4 had the lowest percentage absolute agreement (66.4) and Step 1, the highest (95.9). In Step 4, the choices were ‐ II Failed staff attunement, ‐ I Partially failed staff attunement, + I Partially successful staff attunement and + II Successful staff attunement. The mean score agreement was 92.2%, when combining the ‐ II and ‐ I scores into one category and the + I and + II scores into one category (Step 2 and 4). In Step 4, staff attunement (positive or negative) *and* patient response (positive or negative) to the attunement were scored. Mean score agreement for Step 4 was 89.8%.

## 4 DISCUSSION

The reliability testing of SESPI showed very good results regarding inter‐rater reliability and internal consistency. Cronbach's alpha for the total instrument was 0.98, indicating that raters' scores had a very high internal consistency. ICC was almost 0.80, indicating moderate to good reliability. In general, values above 0.90 are interpreted to indicate excellent reliability and values above 0.70 are considered acceptable for inter‐rater reliability tests (Cicchetti, 1994; Koo & Li, 2016). A commonly accepted rule is that Cronbach's alpha value above 0.7 is acceptable. However Cortina (1993) showed that for scales with many items, the alpha values could be high despite low internal consistency. One of the current study's strengths was that the alpha values were high both for the entire instrument and for each of the four steps separately (each step had 2 to 4 items only). The results of percentage agreement in Table 2 generally support high agreement between the raters. Absolute agreement between raters was 96%, 77% and 84%, respectively, for the three first steps. Step 4 had the lowest agreement (66%). However, almost nine out of 10 raters agreed on whether the excerpt depicted a negative (‐ II or ‐ I) or a positive (+ I or + II) attunement in this step. Thus, a 66% absolute agreement in Step 4 reflects only minor distinctions between the raters' interpretations of the reported attunement. The overall high ICC values indicate that the instrument is sustainable.

The purpose of our study was to develop an instrument for a subsequent quantitative study with scoring of a larger sample of randomized progress notes. The inter‐rater reliability results indicate that the SESPI is a reliable tool for quantitative research. Though systematic reviews have revealed inaccuracy and inadequate documentation of nursing care in general, staff approaches aiming to facilitate therapeutic relationships were not investigated in any of these studies (Müller‐Staub, Lavin, Needham, & Van Achterberg, 2006; Saranto et al., 2014; Wang et al., 2011). To our knowledge, SESPI is the first instrument developed to assess staff–patient interactions in nursing documentation using a quantitative design. This evaluation of nursing documentation takes a different approach than previous studies.

Researchers using the SESPI can discover what percentage of staff progress notes describes staff–patient interactions, as well as get an overview of documented examples of both successful and failed attempts at attunement.

There are many reasons for examining staff–patient interactions from this perspective. First, patients from acute psychiatric units have demanded more empathic understanding from staff (Bee et al., 2008; Hopkins, Loeb, & Fick, 2009; Moreno‐Poyato et al., 2016). Stewart et al. (2015) concluded that initiatives to improve patients' experiences in these contexts were urgently needed. Second, Papadopoulos et al. (2012) found that staff–patient interactions were the most frequent antecedents to violent incidents in psychiatric inpatient settings. Staff's communication strategies in tense situations, for instance regarding limit‐setting, were often found to be ineffective in preventing conflicts from escalating (Bowers et al., 2013; Quanbeck et al., 2007). Step 2 in SESPI assesses descriptions of patients' affects in specific situations. Getting a sense of a patient's affect, whether directly communicated or unspoken, is an essential part of attunement (Delaney et al., 2017). Step 4 may provide data for evaluation of staff–patient interactions regarding approaches proving effective in attuning to the actual patient in actual challenging situations. Thus, evaluation using the SESPI may contribute to a better understanding of which interactions the patient experienced as empathic and, in turn, increase and improve therapeutic staff–patient interactions. Of course, nursing documentation does not necessarily reflect the real experience of the patient. Other sources, such as patient interviews and observation of staff–patient interactions, will be valuable means for making improvements at the individual level. When the goal is to obtain a bigger picture of documentation of interactions at a ward or a hospital, however, the SESPI may be a better alternative.

Nursing staff with empathy ratings above average have been significantly associated with a reduced use of seclusion and restraint in psychiatric inpatient units (Yang, Hargreaves, & Bostrom, 2014). However, little is known about how empathy is developed and maintained in demanding staff–patient relations in acute mental health services (Gerace et al., 2018; Stewart et al., 2015). Delaney et al. (2017) noted that nurses lacked a language to explain how they facilitate therapeutic relationships and depicted a model for practice with attunement and empathy as central elements. SESPI's two categories of successful attunements contain situations where the staff succeeded in attuning to the patient. The patient's responses indicated that he/she had experienced an emphatic understanding in these situations. At the same time, situations that scored in the two categories of failed attunements represented interactions where the patient experienced a lack of understanding from the staff, even though the staff might have had the best intentions. Could SESPI be a tool used for teaching purposes in nursing documentation and would such training support nurses in articulating their efforts to facilitate therapeutic relationships with their patients?

### 4.1 Limitations

The current tests of the scale's reliability were chosen because three out of four steps in the scale consist of 4‐point continuous scales and thus SESPI (as a whole) is considered to be an instrument with continuous variables. Step 1 is the only one without a continuous score scale. Statistically, it might have been better to have had absolute zero score scales for each of the four steps. As described in 2.2, we attempted to use a 5‐point scale, with a zero score reflecting a Neutral experience in Step 2. Similarly, a score point of Neutral attunement was included as one of five categories in Step 4. However, during the first testing of these score categories, we had difficulties operationalizing the Neutral categories and, even worse, finding excerpts that fit with these categories. In our opinion, removing the zero score category in Steps 2 and 4 strengthened the construct validity and the internal validity of the SESPI.

The 10 excerpts for testing the SESPI were picked for having the scope needed to address all four of the steps in the instrument. The content accuracy of the 1051 excerpts varied. We tried to select excerpts with acceptable accuracy and clinical relevance. However, an optimal design would use randomized excerpts and a higher number to be rated. Thus, the next step may be to test inter‐rater reliability with a randomized sample of excerpts.

The results from this research must be interpreted with caution due to the preliminary, exploratory design. The findings may not be directly generalizable to staff in mental health services with different educational backgrounds. A scale's reliability is not a fixed quality, but depends on the specific group of raters and their training in the use of the scale (Streiner & Kottner, 2014). Clearly, when the SESPI is used in research projects or for internal evaluation purposes, new reliability tests are required for each project. This does not mean that our reliability testing of the SEPSI is superfluous. Any development of a new test or instrument requires reliability and validity testing.

## 5 CONCLUSIONS

The results of the reliability tests conducted on the SESPI preliminary support its usefulness for quantitative research. The SESPI is an instrument that can be used to measure the quantity and quality of described nurse–patient interactions. The SESPI measures staff–patient interactions from a self psychology attunement perspective. It includes the extent and quality of reported patient experiences, staff's approaches and whether approaches succeeded in meeting patients' emotional needs. Thus, SESPI is an instrument that can evaluate whether recorded staff–patient interactions were reported as therapeutic. Consequently, quantitative studies using the SESPI may provide data for evaluating nursing documentation in relation to core values in mental health care. It is not known how mental health practice corresponds to what is documented. Hence, it is important for future research to design studies that compare qualitative and quantitative observations of staff–patient interactions with progress notes from the same interactions. Finally, it is paramount to explore how reported interactions correspond to patients' understanding and perception of these interactions.

## CONFLICT OF INTEREST

No conflict of interest has been declared by the authors.

## ETHICAL STATEMENTS

The Regional Committee for medical and health research ethics in Norway approved the study (reference number 2015/1471).
