# Supplementary material for: Development and reliability testing of the Scale for the Evaluation of Staff‐Patient Interactions in Progress Notes (SESPI): An assessment instrument of mental health nursing documentation
Source: Nurs Open. 2019 Mar 21;6(3):790–8. doi: 10.1002/nop2.254 (PMC6650683; doi:10.1002/nop2.254)
Supplement: Supplementary file 1 [file NOP2-6-790-s001.docx]

*Scale for the Evaluation of Staff-Patient Interactions (SESPI)*

An instrument to evaluate

progress notes in nursing documentation

in mental health services

© Molde University College 2019 Kjellaug K. Myklebust & Stål Bjørkly


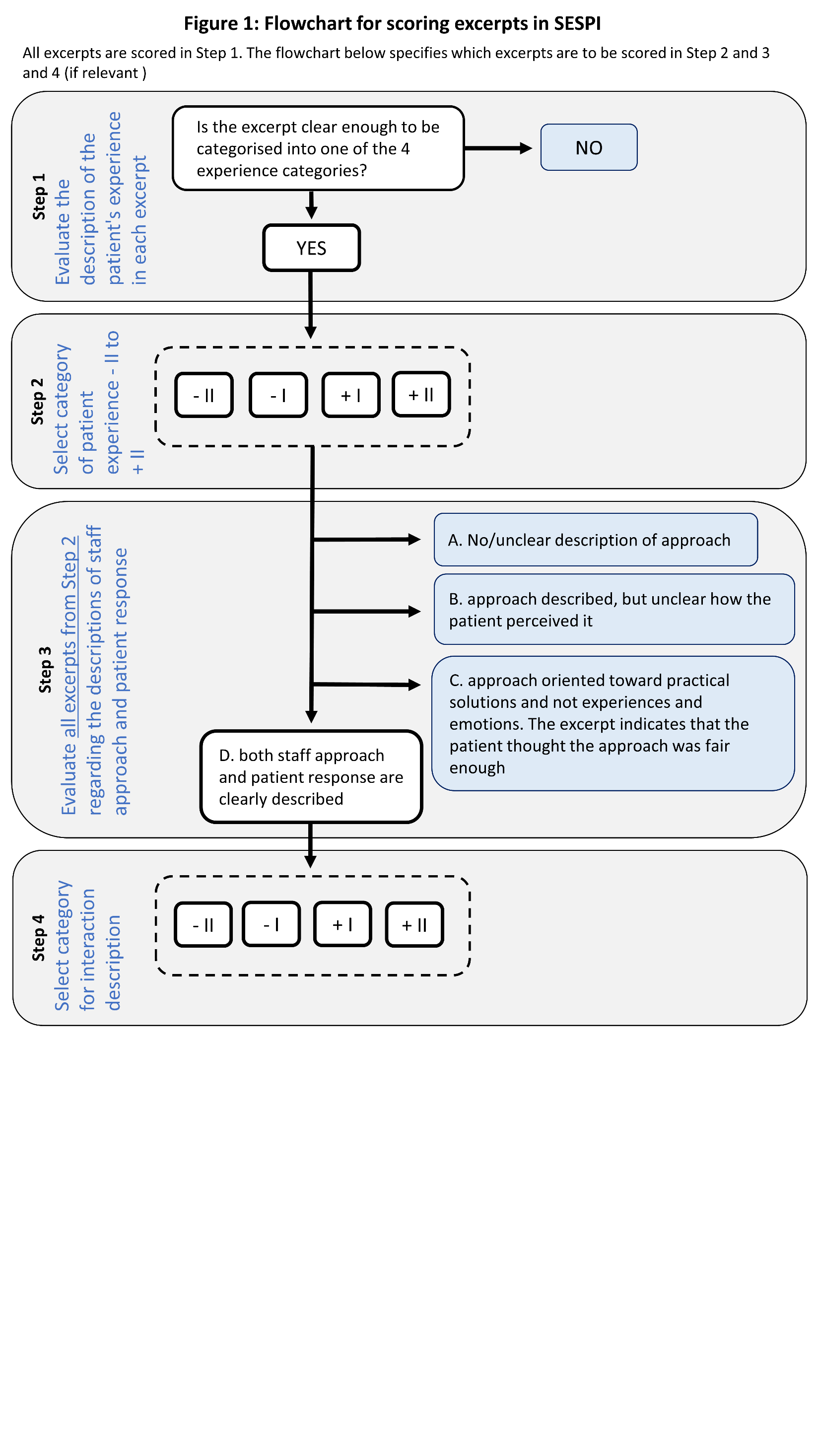


Excerpt = a described episode in a progress note where there was an opportunity for staff and patient to interact.

**STEP 1**

**Evaluate quality of the description of patient’s experience**

Is the patient's experience described adequately enough to be categorised into one of the four categories in Step 2 (see pages 4-7), from very uncomfortable (- II) to very positive (+ II)?

- **Yes.** One of the following points is true:
  - the patient's discomfort is sufficiently clearly described that it can be categorised from

- I to - II.

- - the patient’s positive experience is sufficiently clearly described that it can be categorised from + I to + II.

**If Yes: Go to Step 2 and fill in the appropriate category.**

- **No.** No description of the patient's experience or description insufficient for categorisation.

**If no, do not proceed to categorise the excerpt in the form.**

**STEP 2**

**The patient's experience (before of any staff approach)**

**Select one category:**

- **- II Very uncomfortable. At least one of the following criteria must be met. The excerpt describes:**
- **that the patient explicitly expresses very or comprehensively uncomfortable feelings/thoughts/experiences**
- **interaction that shows that the patient feels very uncomfortable** (e.g. attacks staff, slams door)
- **the staff** **assesses** **that the patient feels very uncomfortable** (without being confirmed by the patient or specific observations: e.g. *He seems to be very depressed.*)

Examples of subjects an excerpt can describe:

- very disagreeable **delusions and hallucinations**, disassociation, flashbacks and dreams or behaviour associated with psychosis.
- **substance abuse**, addiction, withdrawal: strong negative experiences/consequences.
- **significant other**: conflict/rejection/serious concern/extensive privation.
- **existential difficulties**: lack of hope or meaning of life, considerable uncertainty associated with religiosity, sexual orientation.
- **finances, home, housing, job**: considerable uncertainty/anxiety.
- **self-harm or suicide** issues: serious single event or that related thoughts/behaviour have occupied the patient’s thoughts for an extended period.
- **acting out**: breaking, damaging objects, hyperactivity, naked in public.
- **violence**: threats/behaviour intended to physically harm others.
- **somatic ailments**: intense (extensive insomnia, pain, serious difficulties in connection with food intake, severe somatic diseases).
- serious **difficulties in relation to being admitted** (routines, rules, interacting with other patients or staff, therapy/treatment/medication).
- **anxiety, agitation, restlessness, depression**, depressed mood that influences the patient substantially for a complete shift or totally overwhelmed over a shorter period of time.
- **- I** **Uncomfortable. At least one of the following criteria must be met. The report excerpt describes:**
- **that the patient explicitly expresses uncomfortable feelings/thoughts/experiences**
- **interaction that shows that the patient is uncomfortable** (e.g. asks to be left in peace. Will not respond to questions posed)
- **the staff assesses that the patient feels uncomfortable** (without being confirmed by the patient or specific observations, e.g. *She seemed anxious*)

Examples of subjects an excerpt can describe:

- disagreeable **delusions and hallucinations**, disassociation, flashbacks and dreams or behaviour associated with psychosis are described, but the patient is not significantly absorbed/bothered and can concentrate on other things.
- **substance abuse**, addiction, withdrawal: experiences/consequences are not perceived as being a big problem for the patient (e.g. an episode with substance abuse during the admission period).
- **significant other**: moderate frustrations, quarrels, lack of understanding, privation, but no serious conflict or mainly negative experience of the relationship.
- **existential questions** (uncertainty/turmoil related to such issues as the meaning of life, religiosity, sexual orientation, but not perceived as very uncomfortable).
- **finances, home, housing, job, practical tasks**: related concerns/challenges, but not perceived as being highly uncomfortable at the moment.
- **self-harm problems**, moderate: the patient thinks about or commits self-harm to a mild degree, but can easily concentrate on other things, or this is not described as a significant problem for him/her.
- **somatic ailments**, moderate (e.g. tiredness after a sleepless night, pain, lack of appetite, perception of being overweight).
- **difficulties in relation to being admitted** (routines, rules, interacting with other patients or staff, therapy/treatment/medication).
- observations of **passivity** and assessed to be uncomfortable.
- **emotional problems**, moderate or short-term (boredom, restlessness, loneliness, sadness, irritation, mild/moderate anxiety, depression).
- **+** **I Positive. At least one of the following criteria must be met. The excerpt describes:**
- **that the patient explicitly expresses positive feelings/thoughts/experiences** (these can be descriptions that relate the positive experience at the time and not necessarily a description that the patient generally feels well)
- **interaction that shows that the patient has a positive experience**
- **the staff** **assesses that the patient feels better** (without being confirmed by the patient or specific observations. E.g. *He seemed relieved and quieter after being told that his stay at the ward was being extended.*)

Examples of subjects an excerpt can describe:

- signs that **mental difficulties are less** prominent, without the patient's perception being directly described (smiles more, less latency, initiates activities).
- **significant other**: related positive experiences.
- **practical events** outside the institution: positive experiences related to on leave, organising their own home, financial documents).
- **somatic ailments**: improvement of (satisfaction with last night's sleep, less pain).
- positive experiences associated with what is happening **in the ward** (therapy, medication, activities, relationships with other patients, staff and routines).
- satisfaction with periods of the day, an event or an experience that **today is better than yesterday.**
- **+ II** **Very positive. At least one of the following criteria must be met. The excerpt describes:**
- **that the patient expresses vitality, joy, or confidence in own resources**
- **interaction that shows a positive experience by the patient**
- **the staff** **assesses** **that the patient feels very happy or is improving** (without being confirmed by the patient or specific observations).

Examples of areas an excerpt can describe:

- joy/relief over knowing that **uncomfortable emotions are less** dominant (feelings related to anxiety, depression, psychosis, flashbacks).
- joy/ relief at being able to resume/have better contact **with significant others.**
- joy/ relief at being able to **resume interests and work** they may not have had the energy to maintain in the past.
- joy/ relief at being able to **make decisions** that provide better control over their own life.
- joy/relief that intense/serious **somatic ailments are decreasing.**

**STEP 3**

**Evaluate quality of described interaction**

**The excerpt is evaluated based on how the staff have responded in relation to the described patient experience categorised in Step 2, and how the patient responded to this approach.**

- A. No description of the staff's approach, or the description is unclear. (For example, it says that the patient has been anxious, but nothing about what was done in response). Step 4 is **not** filled out.
- B. Approach is described, but how the patient perceived it is unclear (e.g. *She was worried and anxious and was invited to talk about it*). Step 4 is **not** filled out.
- C. Superficial approach described and a positive experience by the patient is implicit in the description. Relevant approaches: practical help, fulfilment of specific wishes, information, advice and explanations, but with no emotional approach or undertaking being described. Category C has a lot in common with + I ‘Partially successful attunement’ in Step 4. In category C, the patient is met in a limited, ‘everyday’ manner, while in + I, Step 4, the approach is more therapeutic in character, aimed at experiences and feelings (examples of category C: *She was anxious and was offered and accepted an anxiolytic pill*. Or: *He was worried about such things as his finances. He wanted to call a friend to borrow money. He was advised to wait until he felt a little better. He accepted this*. Or: *She was stressed and wanted to discuss many things, was advised to lie down and wait until the next day; she lay down and fell asleep quickly*). Step 4 is **not** filled out.
- D. Both the approach from the staff and the patient's response are described in such a way that it can be judged whether the patient felt satisfied or not.

**If D, go to Step 4**

**STEP 4**

**Description of interaction**

**Select one category:**

- **- II Failed attunement.**

**Contingent on description of** **both of the following:**

- **approach from staff that does not show attempts to understand the patient (e.g. patient ordered to be secluded in his room without further explanation)**
- **and the patient clearly perceives the approach as negative**

Examples of category - II interaction:

- **authoritarian correction** with a clear expectation/requirement that the patient will change behaviour or perception and the patient reacts with objection or withdrawal. (E.g. *He seemed scared and told me about multiple voices. He shouted loudly and was told to lower his voice for the sake of other patients, whereupon he began to threaten to kill the staff*.)
- staff **emphasise the power imbalance**.
- does **not** let the patient **express his/her opinion**.
- the staff gives the patient a **task/instruction or explanation** and the patient reacts negatively.
- **- I Partially failed attunement.**

**Contingent on description of** **both of the following:**

- **approach from the staff that precludes further interaction despite the fact that it is milder than - II Failed attunement (can even be an attempt to understand the patient)**
- **and the patient withdraws or explicitly responds negatively to the approach, but to a lesser extent than in category - II**

This category can accommodate a wide range of approaches. Examples of category – I interaction:

- **mildly authoritarian approaches**. (E.g. *She says she has a lot of problems and is then encouraged to be with the rest of us, but says she can't and withdraws*.)
- **efforts to understand/reassure** the patient, but she experiences this approach negatively or the approach precludes further dialogue/interaction. (E.g. *He was invited to talk about the voices, but said he preferred to be alone*.)
- approaches directed only **against part of what the patient conveys**, but significant elements are left unaddressed, which is perceived negatively.
- **+ I Partially successful attunement.**

**Contingent on description of** **both of the following:**

- **approach from staff who seems to be trying to understand/explore/confirm the patient's experience with words or actions, but the content of the approach is inadequately described**
- **and a positive experience from the patient concerning this approach, or that the approach made further positive interaction possible (e.g. the patient began to describe more about his situation)**

Examples of category + I interaction:

- The approach primarily seeks to explore the practicality of what the patient is trying to convey. Even if the approach is not clearly goal-oriented to explore the patient’s emotions, **the patient opens up in response.**
- **invitation to a talk as a response** to staff’s experience that the patient is in trouble/has perceived something positive, but that **the approach in the consultation is not described**. (E.g. *He was troubled after dinner. He agreed to talk it over and says it helps to talk about his thoughts*.)
- offers **physical presence** or to do something with the patient as a response to finding that a patient is in trouble. (E.g. *She said she was troubled by painful memories. She was offered a walk with the staff, and said afterwards that she would like to repeat the walk tomorrow*.)
- actions to **meet the patient’s physical need** and the patient’s subjective experience in the situation.
- compliments, praise, **positive affirmation** regarding something the patient relates/has done, and that the patient seems happy about.
- **+ II Successful attunement.**

**Assumes that the excerpt describes** **both of the following:**

- **approach from the staff which is clearly oriented towards trying to understand/tackle/verify the patient's experience with words or actions**
- **and the patient perceives this approach as a positive experience, or clearly opens up for further interaction**

Examples of category + II interaction:

- **emotional/experience-oriented questions** (how are you?/does it feel?) and that **the patient elaborates** on her experiences. (E.g.: *She shouted and was angry at the staff and said we did not understand. After a while, the contact person went in to her room and asked if it was okay for her to talk. She accepted, and was encouraged to talk more about what it’s like to not to be understood and how she wants to be understood. She then talked about several episodes in which she felt misunderstood, and says she never dared say anything about it in the past.)*
- statement to **confirm the patient's experience** (I understand, I see). (E.g. *He told about an argument with a neighbour, describing the considerable unpleasantness of the experience, and wondered if the contact nurse thought him crazy. She said that it was not difficult to imagine that she would also experience the feelings he described. He seemed relieved over this and went on to talk more about the experience of being crazy.)*
- **attentive action** in response to finding that a patient is in trouble. (E.g. *She had broken a light bulb and was about to harm herself. The contact person asked if he could remove the fragment of glass from the broken bulb. She nodded. After he had removed the fragments, he sat down with her. By the end of the shift, she said that it was a great victory to be able to resist the urge to self-harm*.)
